# An overview of key pretreatment processes for biological conversion of lignocellulosic biomass to bioethanol

**DOI:** 10.1007/s13205-015-0279-4

**Published:** 2015-02-03

**Authors:** Devendra Prasad Maurya, Ankit Singla, Sangeeta Negi

**Affiliations:** 1 Department of Biochemistry and Biochemical Engineering, Sam Higginbottom Institute of Agriculture, Technology and Sciences, Allahabad, 211-007 Uttar Pradesh India; 2 Department of Microbiology and Fermentation Technology, Sam Higginbottom Institute of Agriculture, Technology and Sciences, Allahabad, 211-007 Uttar Pradesh India; 3 Department of Biotechnology, Motilal Nehru National Institute of Technology, Allahabad, 211-004 Uttar Pradesh India

**Keywords:** Lignocellulose, Pretreatment, Agricultural waste, Forest residues, Bioethanol

## Abstract

Second-generation bioethanol can be produced from various lignocellulosic biomasses such as wood, agricultural or forest residues. Lignocellulosic biomass is inexpensive, renewable and abundant source for bioethanol production. The conversion of lignocellulosic biomass to bioethanol could be a promising technology though the process has several challenges and limitations such as biomass transport and handling, and efficient pretreatment methods for total delignification of lignocellulosics. Proper pretreatment methods can increase concentrations of fermentable sugars after enzymatic saccharification, thereby improving the efficiency of the whole process. Conversion of glucose as well as xylose to bioethanol needs some new fermentation technologies to make the whole process inexpensive. The main goal of pretreatment is to increase the digestibility of maximum available sugars. Each pretreatment process has a specific effect on the cellulose, hemicellulose and lignin fraction; thus, different pretreatment methods and conditions should be chosen according to the process configuration selected for the subsequent hydrolysis and fermentation steps. The cost of ethanol production from lignocellulosic biomass in current technologies is relatively high. Additionally, low yield still remains as one of the main challenges. This paper reviews the various technologies for maximum conversion of cellulose and hemicelluloses fraction to ethanol, and it point outs several key properties that should be targeted for low cost and maximum yield.

## Introduction

The energy crisis in the early 1970s enforced research and development aimed at sustainable production of biofuels and chemicals from renewable lignocellulosic feedstocks of agriculture and forestry. Lignocellulosic materials comprise a large fraction of municipal solid waste, crop residues, animal manures, forest residues and dedicated energy crops, also providing the required attributes for reducing greenhouse gases emission (Wyman and Hinman 1990; Wang et al. 1999; Sánchez and Cardona 2008). Moreover, biofuel byproducts could also be utilized as soil amendments which can reduce demands of chemical fertilizers (Singla and Inubushi 2014; Singla et al. 2013, 2014a, b).

Lignocellulosic biomass is composed of cellulose, hemicellulose, lignin, extractives and several inorganic materials, and compositions of each vary depending on the origin of the lignocellulosic material (Singla et al. 2012; Saini et al. 2014). Cellulose is a linear, crystalline polymer of β-d-glucose unit, and the structure is rigid and difficult to break (Chesson and Forsberg 1988). This cellulosic fraction can be converted into glucose by enzymatic hydrolysis or by chemical methods (Mosier et al. 2005). Hemicellulose is composed of linear and branched heteropolymers of d-xylose, l-arabinose, d-galactose, d-glucose and d-mannose. The structure is not crystalline and is, therefore, easier to hydrolyse (Chang and Holtzapple 2000). Lignin is a three-dimensional polymer molecule consisting of three different phenyl-propane precursor monomer units which are particularly difficult to biodegrade. Hence, lignin is the most non-biodegradable component of the plant cell wall (Palonen 2004).

**Fig. 1:**
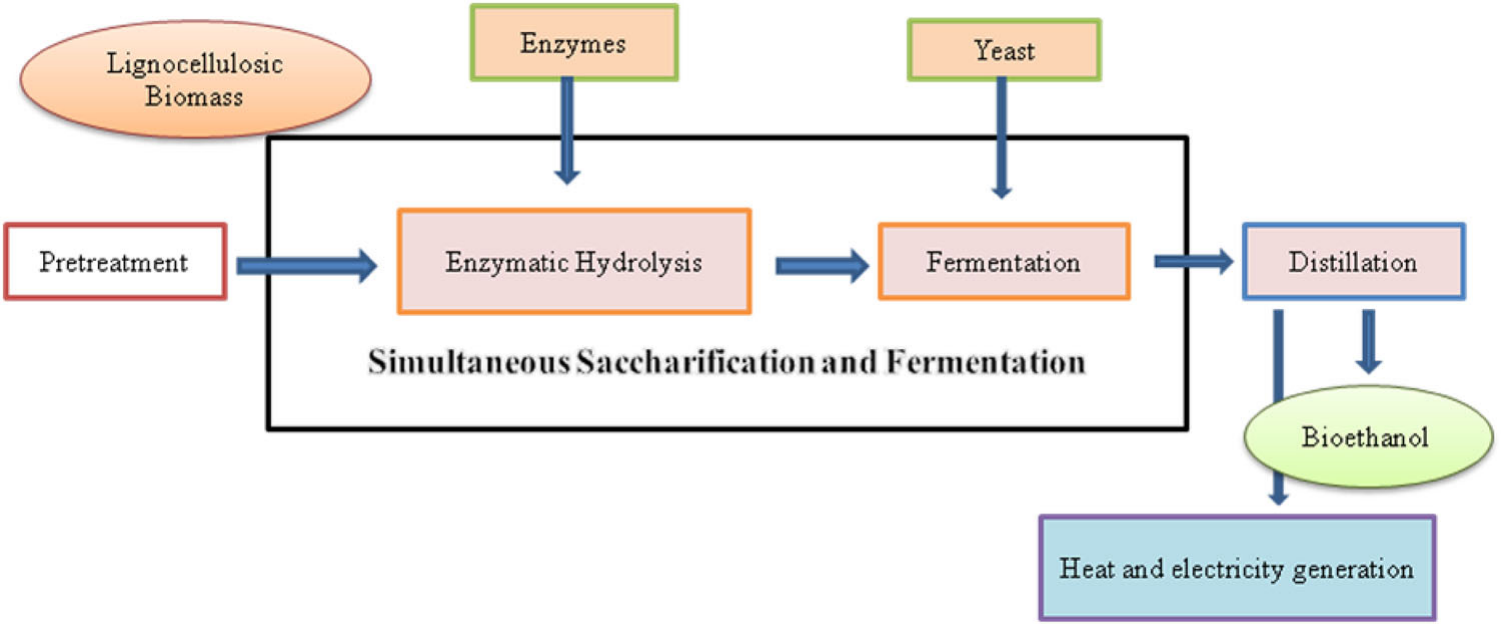
Biological conversion of lignocellulosic biomass to bioethanol

The conversion of lignocellulosic biomass to ethanol comprises the following main steps: hydrolysis of cellulose and hemicellulose to fermentable reducing sugars, fermentation of sugars to ethanol, separation of lignin residue, and finally, recovery and purification of ethanol to meet fuel specifications (Fig. 1). The hydrolysis is usually done by lignocellulosic enzymes and the fermentation is carried out by yeasts or bacteria (Singla et al. 2011; Maurya et al. 2012). The factors that have been identified to affect the hydrolysis of cellulose and hemicelluloses include porosity (accessible surface area) of the waste materials, crystallinity of cellulose, degree of cellulose and hemicellulose polymerization, and degree of acetylation of hemicellulose (Kumar and Wyman 2009a, b). Several pretreatment approaches have been investigated on different varieties of lignocellulosic biomass and theses have shown varying results based on raw material used for fermentation (Carvalheiro et al. 2008; Taherzadeh and Karimi 2008; Yang and Wyman 2008; Alvira et al. 2010; Geddes et al. 2011). It is because of different physico-chemical properties of various lignocellulosic materials. The aim of the effective pretreatment of lignocellulosic biomass should be focused on: (a) increase the accessible surface area and decrystallize cellulose, (b) partial depolymerization of cellulose and hemicellulose, (c) solubilize hemicelluloses and/or lignin, (d) modify the lignin structure, (e) maximize the enzymatic digestibility of the pretreated material, (f) minimize the loss of sugars, and (g) minimize capital and operating costs. An effective pretreatment must also preserve the pentose (hemicellulose) fractions, avoid the need for reducing the size of biomass particles, and limit the formation of toxic components which inhibit growth of fermentative microorganism (Alvira et al. 2010).

In this review, we have emphasized on some of the major and widely used physical, physico-chemical, chemical and biological pretreatment processes of various lignocellulosic biomasses aiming at removal of lignin and conversion of cellulose and hemicellulose into reducing sugars for the production of bioethanol or other value added products. We have discussed the main principles behind these pretreatment processes, their mechanisms, merits/demerits and maximum yield of obtained sugars. The objective of the present review is on the pretreatment processes and recent advances for bioethanol production from different lignocellulosic biomass, and to analyze the interrelated factors between pretreatment, hydrolysis and fermentation.

## Parameters for effective pretreatment of lignocellulosic biomass

There are several key factors which affect the rate of biological degradation of lignocelluloses (Kumar and Wyman 2009b). The accessible surface area for enzymatic attack may be related to cellulose crystallinity, lignin, and hemicellulose content.

### Cellulose crystallinity

The cellulose microfibrils have both crystalline and amorphous regions, and cellulose crystallinity has been considered as one of the important factors in determining the hydrolysis rates of relatively refined cellulosic substrates. The maximum part of cellulose (around 2/3 of the total cellulose) is in the crystalline form (Chang and Holtzapple 2000). In fact, cellulase readily hydrolyzes the more accessible amorphous portion of crystalline cellulose; while the enzyme is not so effective in degrading the less accessible crystalline portion. It is, therefore, expected that high-crystallinity cellulose will be more resistant to enzymatic hydrolysis, and it is widely accepted that decreasing the crystallinity will increase the digestibility of lignocelluloses (Kumar and Wyman 2009b). However, it is not the only factor in effective enzymatic hydrolysis of lignocellulosic biomass due to the heterogeneous nature of celluloses and the contribution of other components such as lignin and hemicelluloses (Kumar and Wyman 2009a).

### Effect of accessible surface area

Studies have indicated a good correlation between the pore volumes (accessible surface area for cellulase and hemicellulase) and the enzymatic digestibility of lignocellulosic materials (Chandra et al. 2007). The main advantage of this correlation is in the improvement of enzymatic hydrolysis by removing lignin. Lignocellulosic biomass has two types of surface area: external and internal. The external surface area is related to the particle size and shape; while the internal surface area depends on the capillary structure of cellulosic fibers.

### Effect of lignin

The presence of lignin is responsible for integrity, structural rigidity and the prevention of swelling of lignocellulosic material. The cellulose and hemicellulose are covered by lignin. The presence of lignin hinders the access of enzymes to cellulose and hemicelluloses (Kumar and Wyman 2009b), thus reducing the efficiency of the hydrolysis. Lignin is the most important recognized factor for recalcitrance of lignocellulosic materials. Therefore, efficient delignification processes can improve the rate and extent of enzymatic hydrolysis (Laureano-Pérez et al. 2005)

### Effect of hemicellulose

Hemicellulose is a physical barrier which covers the cellulose fibers and protects it from the enzymatic hydrolysis. It has been shown that the removal of hemicellulose increases the mean pore size of the substrate and, therefore increases the accessibility and the probability of the cellulose hydrolysis (Jeoh et al. 2005; Chandra et al. 2007; Ishizawa et al. 2007). Degree of acetylation in the hemicellulose is another important factor as lignin and acetyl groups are attached to the hemicellulose matrix and may hinder polysaccharide breakdown (Chang and Holtzapple 2000).

## Pretreatment of lignocellulosic biomass

**Fig. 2:**
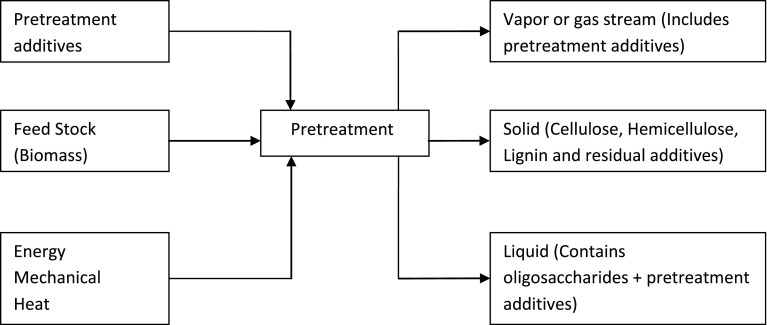
Design and economic viability of pretreatment technologies of lignocellulose for bioethanol production

Various pretreatment methods are now available to fractionate, solubilize, hydrolyze and separate cellulose, hemicellulose, and lignin components (Fig. 2). These include concentrated acid, sulfur dioxide (SO_2_), hydrogen peroxide (H_2_O_2_), steam explosion (autohydrolysis), ammonia fiber expansion (AFEX), wet oxidation, lime, liquid hot water, carbon dioxide (CO_2_) explosion and organic solvent treatments (da Costa et al. 2009; Karimi et al. 2013). The Physical (mechanical), physico-chemical, chemical, and biological processes have been used for pretreatment of lignocellulosic materials. Mechanical pretreatment increases the surface area by reducing the size of the biomass. A high control of operating conditions is required in the physico-chemical methods because these reactions occur at high temperature and pressure (Taherzsadeh and Karimi 2007). Chemical methods remove and/or dislocate hemicelluloses and lignin and thus, loosening the structural of lignin holocellulose network. Biological pretreatment methods are used for the delignification of lignocellulosic biomass (Chandel et al. 2007).

### Physical pretreatments

#### Mechanical comminution

The objective of the mechanical pretreatment is to reduce the particle size and crystallinity of lignocellulosic materials to increase the specific surface area, and to reduce the degree of polymerization of cellulose. The size of feedstock materials is usually 10–30 mm after chipping and 0.2–2 mm after milling or grinding (Sun and Cheng 2002). Different milling processes (two-roll milling, hammer milling, colloid milling and vibratory milling) can be used to improve the digestibility of the lignocellulosic materials compared to ordinary ball milling (Taherzadeh and Karimi 2008). This process is generally not economically feasible because of high energy consumption for obtaining desired particle size (Zhu and Pan 2010).

#### Extrusion

Extrusion process has been used to produce gaseous products and residual char (Shafizadeh and Bradbury 1979). In this process, materials are treated at a temperature higher than 300 °C followed by mixing and shearing which results in physical and chemical modifications of cellulose. The screw speed and barrel temperature are believed to disrupt the lignocellulosic structure causing defibrillation, fibrillation and shortening of the fibers, followed by increased arability of carbohydrates to enzymatic attack (Karunanithy et al. 2008). The various parameters in bioreactor must be highly efficient in this process. In recent study, the application of enzymes during extrusion process is considered as a novel technology for ethanol production (Zheng et al. 2014).

### Physico-chemical pretreatments

#### Steam explosion (autohydrolysis)

Steam explosion is the most commonly used method for pretreatment of lignocellulosic biomass (Chandra et al. 2007; Singh et al. 2015). In this method, the chipped biomass is treated to high pressure saturated steam for few seconds (30 s) to several minutes (20 min), and then pressure is suddenly reduced. Steam explosion is typically a combination of mechanical forces and chemical effects due to the hydrolysis (autohydrolysis) of acetyl groups of hemicellulose. Autohydrolysis is initiated at high temperatures (160–260 °C) which promote the formation of acetic acid from acetyl groups (Pan et al. 2005; Quievy et al. 2009). Furthermore, water can also act as an acid at high temperatures. The mechanical effects are caused because of sudden reduction in pressure and fibers are separated owing to the explosive decompression. This process causes hemicellulose degradation and lignin transformation due to high temperature, thus increasing the potential of cellulose hydrolysis (Pan et al. 2005). The most important parameters that affect the steam explosion pretreatment are: particle size, temperature, residence time, moisture content and the combined effect of temperature (*T*) and time (*t*), which is defined by the severity parameter (*Ro*) [*R*o = *t* exp ^[T−100/14.75]^: *t* is the reaction time (min), and *T* is the hydrolysis temperature (°C)]. The optimal conditions for maximum sugar yield following severity parameter were found 3.0–4.5 (Alfani et al. 2000).

Steam explosion processes have many attractive features compared to other pretreatment technologies (Table 1). These include the potential for significant improvement in enzymatic hydrolysis, lower environmental impact, lower capital investment, more potential for energy efficiency, less hazardous process chemicals and conditions, and high sugar recovery (Avellar and Glasser 1998). The advantage of steam explosion pretreatment also includes the possibility of using larger chip size, avoiding unnecessary addition of acid catalyst (except for softwoods), and its feasibility at industrial scale. Steam explosion is recognized as one of the most effective processes for cost reduction in hardwoods and agricultural residues, but it is less effective for softwoods because of low content of acetyl groups in the hemicellulosic portion of softwoods (Sun and Cheng 2002). The addition of SO_2_ or sulfuric acid (H_2_SO_4_) has been proposed as one of the most effective pretreatment methods for softwood material but it has some disadvantages (Berlin et al. 2006; Kumar et al. 2012). The main drawback of this process is equipment requirement for acid addition and the formation of inhibitory/degrading compounds (Mosier et al. 2005).

**Table 1:** Advantages and disadvantages of different pretreatment methods of lignocellulosic biomass

Pretreatment method	Advantages	Disadvantages
Milling	-Decrease of cellulose crystallinity and degree of polymerization -Reduction of particle size to increase specific surface area and pore size	-High power and energy consumption
Steam explosion	-Causes lignin transformation and hemicellulose solubilization -Lower cost -Higher yield of glucose and hemicellulose in the two-step method	-Generation of toxic compounds -Partial hemicellulose degradation
Liquid hot water	-Size reduction of the biomass is not needed -No chemicals are generally required -No requirement of corrosion-resistant materials	-High energy and high water requirement -Formation of toxic compounds
Ammonia fiber expansion (AFEX)	-Increases accessible surface area -Less inhibitors formation -Does not require small particle size of biomass	-Not very effective for the biomass with high lignin content -High cost of large amount of ammonia
CO_2_ explosion	-Increase accessible surface area -Availability at relatively low cost -Do not form inhibitory compounds -Non-flammability -Easy recovery after extraction and environmental acceptability	-Very high pressure requirements
Wet oxidation	-High degree of solubilization of hemicellulose and lignin -Avoid formation of degradation compounds	-High cost of oxygen and alkaline catalyst
Concentrated acid	-High glucose yield -Ambient temperatures	-High cost of acid and need to be recovered -Corrosion-resistant equipments are required -Concentrated acids are toxic and hazardous
Diluted acid	-High recovery of sugars at the end of the process -Low formation of toxic products	-Concentration of reducing sugars is relatively low -Generation of degradation products
Alkali	-Decrease in the degree of polymerization and crystallinity of cellulose -Disruption of lignin structure	-High cost -Not used for large-scale plant
Ozonolysis	-Effectively removes lignin content -Does not produce toxic residues -Reaction is carried out at room temperature and pressure	-High cost of large amount of ozone
Organosolv	-Causes lignin and hemicellulose hydrolysis	-Solvents need to be drained and recycled -High cost
Biological	-Low energy requirements -Delignification -Reduction in degree of polymerization of cellulose -Partial hydrolysis of hemicelluloses -No chemical requirements -Mild environmental conditions	-Slow process rate -Very low treatment rate -Not very effective for commercial application

The main drawbacks of steam explosion pretreatment are the partial degradation of hemicelluloses and the formation of toxic components that could affect the enzymatic hydrolysis and fermentation process (Oliva et al. 2003). Another drawback is the energy consumption for obtaining final chip size before pretreatment which can make up one-third of the power requirement of entire process (Hamelinck et al. 2005). Steam explosion and acid hydrolysis pretreatment may sometime produce furfural and hydroxymethylfurfural as byproducts which have inhibitory effect on the fermentation process. Hence, a separate detoxification step (e.g., addition of activated charcoal, over liming, ion exchange) becomes necessary, thereby increasing the overall process cost (Schmidt and Thomsen 1998; Yang and Wyman 2008).

#### Liquid hot water

Liquid hot water is one of the hydrothermal pretreatment which does not require rapid decompression and the addition of any catalyst or chemicals. Water pretreatment under high pressure is used to maintain the water in the liquid state at elevated temperatures. Temperature range between 170 and 230 °C and pressure (>5 MPa) are commonly used (Sánchez and Cardona 2008). Liquid hot water removes hemicellulose from lignocellulosic materials which makes the cellulose more accessible (Table 1). The obtained slurry after pretreatment can be filtered to obtain two fractions: one solid cellulose-enriched fraction and a liquid fraction rich in hemicellulose derived sugars. This pretreatment allows better pH control (4–7) which minimizes the non-specific degradation of polysaccharides and also avoids formation of inhibitors (Mosier et al. 2005). Liquid hot water has shown the potential to release high fraction of hemicellulosic sugars mostly in the form of oligomers contributing to reduce the undesired degrading products (Mosier et al. 2005). It was reported that temperature and time showed the most significant effect on the recovery of hemicellulosic sugars and the yield of subsequent enzymatic hydrolysis of pretreated corn stover (Mosier et al. 2005), sugarcane bagasse (Laser et al. 2002) and wheat straw (Perez et al. 2008). Three methods have also been developed to promote an effective contact between the biomass and the liquid water: co-current, countercurrent, and flow-through. In co-current pretreatments, slurry of biomass and water is heated to the desired temperature and held at the pretreatment conditions for controlled residence time before being cooled. Countercurrent pretreatment is designed to move water opposite to lignocellulose through the pretreatment system. Flow-through system allows hot water passage over a stationary bed of lignocelluloses; which hydrolyzes and dissolves lignocellulose components and carries them out of the system (Liu and Wyman 2003; Yang and Wyman 2004).

In general, liquid hot water pretreatments are attractive from a cost savings point of view because no chemicals and corrosion-resistant materials are required for hydrolysis reactors. Another major advantage is that the solubilized hemicellulose and lignin products are present in lower concentration due to high water input (Table 1). Higher pentose recovery and lower formation of inhibitory components are obtained in this pretreatment compared to steam explosion. However, this process is yet not developed at commercial scale because of higher water demand and high energy requirement.

#### Ammonia-based pretreatments

AFEX is another type of physico-chemical pretreatment in which lignocellulosic biomass is treated with liquid ammonia at relatively moderate temperature (90–100 °C) for a period of 30–60 min. followed by a rapid pressure release (Kim et al. 2011). It results in a rapid expansion of the liquid ammonia that causes swelling and physical disruption of biomass fibers and partial decrystallization of cellulose. AFEX produces only a pretreated solid material. AFEX process can either modify or effectively reduce cellulose crystallinity and lignin fraction of the lignocellulosic materials (Laureano-Pérez et al. 2005). AFEX increases the digestibility of lignocellulosic biomass by removing the least acetyl groups by deacetylation process (Kumar and Wyman 2009a, b). The main advantage of the ammonia pretreatment is that it does not produce inhibitors for the downstream biological processes, so water wash is not necessary (Table 1). The herbaceous and agricultural residues are more effective for AFEX pretreatment, with limited effectiveness on woody biomass and other high lignin feedstocks (Wyman et al. 2005a). Shao et al. (2010) demonstrated that AFEX pretreated corn grain yielded 1.5–3.0 folds higher enzymatic hydrolysis compared to untreated substrates. Sequential addition of cellulases after hydrolysis of starch resulted in 15–20 % higher hydrolysis yield compared to simultaneous addition of hydrolytic enzymes. AFEX pretreated corn stover resulted in 70 % glucan conversion after 72 h of hydrolysis. Ethanol fermentation of AFEX treated (at 6 % w/w glucan loading) corn stover resulted in 93 % ethanol yield (Uppugundla et al. 2014). (Teymouri et al. 2005) optimized the conditions such as ammonia loading, temperature, blowdown pressure, moisture content of biomass and residence time in the AFEX process. It has been observed that at optimal conditions, AFEX can achieve over 90 % conversion of cellulose and hemicellulose to fermentable sugars for a broad variety of lignocellulosic materials. The high volatility of ammonia allows it to be recovered and recycled, leaving the dried biomass ready for enzymatic hydrolysis (Sendich et al. 2008).

The main disadvantage of AFEX process is that it is more effective on the biomass that contains less lignin (Table 1). Furthermore, ammonia must be recycled after the pretreatment to reduce the cost and protect the environment (Sun and Cheng 2002). The cost of ammonia recovery may be significant regarding the commercial potential of the AFEX pretreatment (Mosier et al. 2005). Another type of ammonia-based methodology is ammonia recycled percolation (ARP) in which aqueous ammonia (5–15 wt %) passes through a packed bed reactor along with biomass at elevated temperature (140–210 °C) for 90 min, and percolation rate is kept 5 mL/min (Sun and Cheng 2002; Kim et al. 2008). ARP can solubilize hemicellulose and lignin, and both can be removed from the biomass as the liquid phase (Yang and Wyman 2008). An important challenge for ARP is to reduce the liquid loading or process temperature to reduce energy cost. Soaking in aqueous ammonia (SAA) at lower temperatures (40–90 °C) for longer reaction times has been used to preserve most of the glucan and xylan in the samples which is subsequently fermented using the simultaneous saccharification and co-fermentation (SSCF) process (Kim et al. 2008).

#### CO_2_ explosion

This method is based on the utilization of CO_2_ as a supercritical fluid in which fluid displays gas like mass transfer properties besides a liquid-like solvating power. Supercritical pretreatment conditions can effectively remove lignin by increasing enzymatic digestibility of aspen (hardwood) and southern yellow pine (softwood) (Kim and Hong 2001). The delignification with CO_2_ (SC-CO_2_) at high pressure can be improved by the addition of co-solvents such as ethanol. Supercritical CO_2_ has been mostly used as an extraction solvent for non-extractive purposes due to its several advantages such as availability at relatively low cost, non-toxicity, non-flammability, easy recovery after extraction, and the environmental acceptability (Table 1; Schacht et al. 2008). In aqueous solution, CO_2_ forms carbonic acid and increases hydrolysis rate. The size of CO_2_ molecules should be comparable to water and ammonia because CO_2_ molecules can penetrate small pores accessible to water and ammonia molecules. In this pretreatment, disruption of cellulose and hemicellulose structure occurs and consequently accessible surface area of the substrate to enzymatic attack increases. The comparison of CO_2_ explosion with steam and ammonia expansion pretreatment methods on several substrates showed that CO_2_ explosion was more cost-effective than ammonia expansion and the formation of inhibitors was lower compared to steam explosion (Zheng et al. 1998).

#### Oxidative pretreatment

Oxidative pretreatment involves the addition of an oxidizing agent such as H_2_O_2_ or peracetic acid (C_2_H_4_O_3_) to the water-suspended biomass. H_2_O_2_ is the most commonly used oxidizing agent. Studies have shown that dissolution of about 50 % of lignin and most of the hemicellulose has been achieved in a solution of 1–2 % H_2_O_2_ at 25–30 °C (Chaturvedi and Verma 2013). This solubilization is generally five folds higher than those of sodium hydroxide (NaOH) treatment without H_2_O_2_ addition. This pretreatment method removes hemicellulose and lignin from biomass to increase accessibility to the cellulose (García-Cubero et al. 2009). Several reactions like electrophilic substitution, displacement of side chains, cleavage of alkyl/aryl ether linkages or the oxidative cleavage of aromatic nuclei can occur during this pretreatment (Hon and Shiraishi 2001). It has been observed that diluted alkaline peroxide treatment is an effective method for pretreatment of rice hulls, resulting in almost complete conversion (96 %) of rice hulls to sugars after enzymatic hydrolysis (Saha and Cotta 2007).

#### Wet oxidation

Wet oxidation is considered as a suitable process for pretreatment of biomass having high lignin content. In this process, materials are treated with water and air/oxygen at temperatures higher than 120 °C for 30 min (Varga et al. 2004). The temperature, reaction time and oxygen pressure are the most effective parameters in wet oxidation (Schmidt and Thomsen 1998). The addition of oxygen at temperatures higher than 170 °C makes the process exothermic, and it becomes self-supporting system with respect to heat (Schmidt and Thomsen 1998). The wet oxidation pretreatment catalyzes the formation of acids from hydrolytic processes and oxidative reactions. All three fractions of lignocellulosic materials are affected in this process. The hemicelluloses are extensively cleaved to low molecular weight sugars that become soluble in water. Lignin undergoes cleavage and oxidation, and cellulose is partly degraded. The cellulose becomes highly susceptible to enzymatic hydrolysis. However, addition of some alkaline agent such as sodium carbonate may help to solubilize hemicellulose fraction and also minimizes the formation of furan-based degradation products that could inhibit enzymes (Ahring et al. 1996).

Szijarto et al. (2009) showed a solubilization of 51.7 % of the hemicellulose and 58.3 % of the lignin; whereas 87.1 % of the cellulose remained in the solids while studying common reed (*Phragmites australis*). The optimum conditions (185 °C, 12 min) increased the digestibility of reed cellulose more than three times compared to the untreated control. The conversion of 82.4 % cellulose to glucose was also achieved during the same process. Simultaneous saccharification and fermentation of pretreated solids resulted in a final ethanol concentration as high as 8.7 g/L, yielding 73 % of the theoretical yield. Banerjee et al. (2011) investigated pretreatment of rice husk by alkaline peroxide-assisted wet air oxidation (APAWAO) to increase the enzymatic convertibility of cellulose. Rice husk was presoaked overnight in 1 % (w/v) H_2_O_2_ solution at room temperature, followed by wet air oxidation (WAO). APAWAO pretreatment resulted in solubilization of 67 % of hemicellulose and 88 % of lignin. It also resulted in 13 folds increase in the amount of glucose compared to untreated rice husk. Almost 86 % of cellulose was converted into glucose within 24 h. The main advantage of wet oxidation is the formation of less inhibitors and efficient removal of lignin (Table 1). The main drawback of this process is that it requires the maintenance of high temperature and pressure, and the presence of strong oxidizing agents such as H_2_O_2_. These requirements lead to high costs of maintenance and also require large-scale reaction vessels. Therefore, application of this process in large-scale pretreatment of biomass is limited. The cost of oxygen and catalyst are another disadvantages for this process.

#### Microwave pretreatment

Microwave irradiation is a process which has been widely used because of its high heating efficiency and easy operation. The residence time in microwave irradiation ranges from 5 to 20 min. It could change the ultra structure of cellulose by degrading lignin and hemicelluloses and by increasing the enzymatic susceptibility of lignocellulosic materials (Maurya et al. 2013). Preliminary experiments identified alkali-treated rice straw as suitable biomass for microwave-based pretreatment (Zhu et al. 2006). NaOH is the most effective alkali reagent for microwave-based pretreatment. One of the studies on microwave-based alkali pretreatment of switchgrass observed the low energy requirement for extended pretreatment time and obtained 70–90 % sugar yields (Hu and Wen 2008). Xu et al. (2011) developed an orthogonal design to optimize the microwave pretreatment of wheat straw and observed ethanol yield of 148.93 (g/kg wheat straw) which was much higher from untreated material (26.78; g/kg wheat straw). Boonmanumsin et al. (2012) reported substantial increase in monomeric sugars yield of *Miscanthus sinensis* while carrying out microwave-assisted ammonium hydroxide treatment. A loss of 74 % lignin and 24.5 % holocellulose was reported with a yield of 41 % of total reducing sugars in microwave pretreatment of oil palm empty fruit bunch fiber in the presence of alkaline conditions (Nomanbhay et al. 2013).

The main advantage of this process is the short reaction times and homogeneous heating of the reaction mixture. Microwave-assisted pretreatment of biomass could be a useful process to save time, energy and minimum generation of inhibitors. It could be considered as one of the most promising pretreatment methods to change the native structure of cellulose with lignin and hemicelluloses degradation, and thus increasing the enzymatic susceptibility (Lu et al. 2011). Microwave approach could be further combined with the addition of chemicals to improve the sugar yield from the substrate.

### Chemical pretreatments

#### Acid pretreatment

The main objective of the acid pretreatment is chemical hydrolysis which can cause solubilization of hemicelluloses and lignin, and to make the cellulose more accessible to enzymes. Acid pretreatment technologies can be performed with concentrated or diluted acid (Table 1) but use of concentrated acid is less attractive due to the formation of inhibiting compounds (furfural, 5-hydroxymethylfurfural, phenolic acids and aldehydes). Concentrated acids are toxic, corrosive, hazardous, and require equipment that is resistant to corrosion. Diluted acid pretreatment method is the most feasible for industrial scale. Different types of reactors such as percolation, plug flow, shrinking-bed, batch, flow-through reactor and countercurrent reactors have been developed for this approach (Taherzadeh and Karimi 2008). There are two types of dilute acid pretreatment processes: high temperature (e.g., 180 °C) during a short period of time and lower temperature (e.g., 120 °C) for longer retention time (30–90 min). High hydrolysis yields have been reported with dilute H_2_SO_4_ which is also the most widely used acid (Mosier et al. 2005; Sindhu et al. 2011).

However, use of hydrochloric acid (HCl), phosphoric acid, nitric acid, C_2_H_4_O_3_, oxalic acid, formic acid, acetic acid and maleic acid has also been tested (Hernández-Salas et al. 2009; Gámez et al. 2006; Rodriguez-Chong et al. 2004; Lee et al. 2011). Oxalic acid treatment in corn cobs produced low level of inhibitors with a total sugar yield of 13.1 % (Lee et al. 2011); while it was 10 % in maleic acid treatment with the generation of higher levels of furfural and hydroxymethylfurfural (Lee and Jeffries 2011). Kim et al. (2011) carried pretreatment of rice straw in two-stage process using aqueous ammonia and dilute H_2_SO_4_ in percolation mode. The yield of reducing sugars was observed 96.9 and 90.8 %, respectively, indicating that combination of these two processes resulted in better removal of lignin and hemicelluloses. Pretreatment liquor of *Eulaliopsis binata* (a perennial grass commonly found in India and China) with diluted H_2_SO_4_ at optimum conditions resulted in 21.02 % total sugars, 3.22 % lignin and 3.34 % acetic acid with the generation of low levels of inhibitors (Tang et al. 2013). Bondesson et al. (2013) reported 78 % yield in corn stover by following steam pretreatment with diluted H_2_SO_4_. Acid pretreatment of biomass could be inexpensive because H_2_SO_4_ and HCl are cheap (Table 1). The process is carried out at high temperatures, and therefore, it requires high energy input, which is costly. The presence of acids at high temperatures can be corrosive, thus, the process requires specific reaction vessels which must be resistant to these conditions. In addition, acid treatment generates inhibitors which need to be removed.

#### Alkali pretreatments

In the alkaline treatment, biomass is treated with alkali such as sodium, potassium, calcium and ammonium hydroxides at normal temperature and pressure. The main advantage of the process is efficient removal of lignin from the biomass (Table 1). This process removes acetyl and uronic acid groups present on hemicelluloses, thus enhances the accessibility of enzyme that degrades hemicellulose (Chang and Holtzapple 2000). Ester linkages between xylan and hemicelluloses residues are also hydrolyzed (Sun and Cheng 2002). This process can largely improve the cellulose digestibility and it is also more effective for lignin solubilization, exhibiting minor cellulose and hemicellulose solubilization compared to acid pretreatment (Carvalheiro et al. 2008). Alkali pretreatment can also be operational at lower temperature, pressure and time ranging from hours to days. NaOH is more effective than others (Sun et al. 1995; Kumar and Wyman 2009a). It was found to be more effective on increasing the internal surface area of cellulose, decreasing the degree of polymerization and crystallinity, and disrupting the lignin structure (Taherzadeh and Karimi 2008). However, no effect of dilute NaOH was observed on softwoods with lignin content greater than 26 % (Kumar and Wyman 2009a).

Lime [Ca(OH)_2_] is another widely used alkali. It also removes acetyl groups and lignin-carbohydrate ester and enhances cellulose digestibility (Mosier et al. 2005). It has been proven successful for pretreatment of wheat straw, poplar wood, switchgrass and corn stover (Chang et al. 2001; Kim and Holtzapple 2006). This pretreatment has the additional benefits of low reagent cost and less safety requirements compared to NaOH or KOH pretreatments and can be easily recovered from hydrolysate by reaction with CO_2_ (Mosier et al. 2005). The addition of air/oxygen to alkaline pretreatment [NaOH/Ca(OH)_2_] can improve the treatment efficiency by increasing lignin removal (Carvalheiro et al. 2008). Some researchers have also tried combination of two pretreatment processes for significant recovery of reducing sugars: combination of alkaline treatment (lime) with oxidative delignification process. Although, lime and other hydroxides are inexpensive but downstream processing costs are high, thus making it a costly process (Table 1). The process also utilizes a huge amount of water for washing salts of calcium and sodium. Moreover, it is difficult to remove them.

#### Ozonolysis

The biomass is treated with ozone (O_3_) which is a powerful oxidizing agent. It degrades lignin by attacking aromatic rings structures, and does not affect hemicellulose and cellulose. It can be used to disrupt the structure of many lignocellulosic materials such as wheat straw, bagasse, pine, peanut, cotton straw, rye straw and poplar sawdust (Sun and Cheng 2002; García-Cubero et al. 2009). Ozonolysis is usually performed at room temperature and pressure, and it does not produce toxic residues that can affect the subsequent hydrolysis and fermentation. The O_3_ gas is passed through a reaction vessel containing the substrate. The vessel could be packed beds, fixed beds or stirred semi-batch reactors (Vidal and Molinier 1988; García-Cubero et al. 2009).

Moisture content and type of biomass significantly affect ozonolysis. Miura et al. (2012) studied the effect of ozonolysis and wet disk milling (WDM) on Japanese cedar (*Cryptomeria japonica*) to improve sugar production by enzymatic saccharification. They observed decrease in O_3_ consumption if moisture content reached more than 40 % and it resulted in less delignification. The application of WDM following O_3_ treatment increased glucose and xylose yields (68.8 and 43.2 %, respectively) without significantly affecting mannose yield. A major drawback of ozonolysis is the requirement of large amounts of O_3_, making the process expensive (Sun and Cheng 2002).

#### Organosolv

Organosolv process uses organic or aqueous organic solvent mixtures with inorganic acid catalysts to extract lignin from lignocellulosic biomass. Numerous organic solvent mixtures including methanol, ethanol, acetone, ethylene glycol, triethylene glycol and tetrahydrofurfuryl alcohol have been used (Zhao et al. 2009a). Some organic or aqueous organic solvents like oxalic, acetylsalicylic and salicylic acid can also be used as catalysts at higher temperatures with or without addition of some organic acids (Sarkanen 1980). Pretreatment of wheat straw by glycerol-based autocatalytic organosolv pretreatment resulted in removal of 70 % hemicelluloses and 65 % lignin (Sun and Chen 2008). It also resulted in 98 % cellulose retention. A modified organosolv method using ethanol under mild conditions followed by H_2_O_2_ post-treatment in horticultural waste resulted in a hydrolysate containing 26.9 g/L reducing sugar (Geng et al. 2012). Fermentation of this hydrolysate medium produced 11.69 g/L ethanol using *Saccharomyces cerevisiae*. Hideno et al. (2013) have reported that the application of alcohol-based organosolv treatment in combination with Ball Milling (BM) for pretreatment of Japanese cypress (*Chamaecyparis obtusa*) significantly improved the enzymatic digestibility and decreased the required severity of organosolv treatment. It was also observed that the combination of alcohol-based organosolv treatment in mild conditions and short time BM had a synergistic effect on the enzymatic digestibility of Japanese cypress. Organosolv process has been extensively used for extraction of high quality lignin which is a value added product. This process has shown high amounts of enzymatic hydrolysis of treated biomass (around 90 %) due to efficient removal of lignin.

The main drawback of the process is the cost of solvent and the catalysts (Table 1). Removal and recovery of the solvent can considerably reduce the operational cost (Sun and Cheng 2002). Another important aspect is safety measures which have to be implemented because organic solvents are inflammable and uncontrolled use can cause fires and explosions. This additional requirement increases the cost of the process. Organic solvents are also the inhibitors of enzymatic hydrolysis, so their removal is necessary for proper enzymatic hydrolysis (Mosier et al. 2005). Removal of organic solvents also burdens an additional cost.

#### Ionic liquids (ILs)

This pretreatment process uses ILs in a ratio of biomass and ionic liquid (1:10 w/w) and temperatures ranging from 100 to 150 °C. The antisolvent such as water, methanol and ethanol use the regeneration of soluble biomass and then subject to enzymatic hydrolysis to produce fermentable sugars. ILs behave like salt which is typically a combined effect of large organic cations and small inorganic anions and it exist as liquids at relatively low temperatures (room temperature). ILs have the capability to form hydrogen bonds with cellulose at high temperatures because of the presence of anions like chloride, formate, acetate or alkyl phosphonate. ILs have tremendous potential for pretreating lignocellulosic biomass and producing a substrate that can achieve more than 90 % cellulose digestibility (Lee et al. 2009).

Residual ILs remaining in the biomass could interfere with hydrolytic enzyme activities and downstream fermentation steps (Sathitsuksanoh et al. 2012; Shi et al. 2013). It may affect the final sugar and biofuel yields. After regeneration, ILs may be recovered from antisolvents by flash distillation and it could be reused (Joglekar et al. 2007). Development of energy efficient recycling methods for ILs is a prerequisite for large-scale application. Toxicity to enzymes and fermentative microorganisms must also be considered before their application in biomass pretreatment (Yang and Wyman 2008; Zhao et al. 2009b). Significant negative effect on cellulase activity may also occur in ILs treatment. Further research is needed to improve the economics of ILs pretreatment before they can be applied at industrial scale. In addition, techniques need to be developed to recover hemicellulose and lignin from solutions after extraction of cellulose. Despite these current limitations, development of ILs pretreatment offers a great potential for future biorefinering processes of lignocelluloses.

### Biological pretreatments

Conventional physico-chemical methods for lignin degradation require large inputs of energy and also cause pollution. Therefore, biological pretreatment of lignocellulosic biomass is considered as an efficient, ecofriendly and cheap alternative (Wan and Li 2012). The biological pretreatment of lignocellulosic biomass is usually performed using cellulolytic and hemicellulolytic microorganisms. The commonly used microorganisms are filamentous fungi which are ubiquitous and can be isolated from the soil, living plants or lignocellulosic waste material (Vats et al. 2013). Studies have shown that white-rot fungi are the most effective microorganisms for the pretreatment of most of the lignocellulosic materials (Kumar and Wyman 2009a). Several white-rot fungi such as *Phanerochaete chrysosporium*, *Ceriporia lacerata*, *Cyathus stercolerus*, *Ceriporiopsis subvermispora*, *Pycnoporus* *cinnarbarinus*, *Pleurotus* *ostreaus* and *P. chrysosporium* produce lignin peroxidases which is lignin-degrading enzymes and manganese-dependent peroxidases. These have shown high delignification efficiency on various lignocellulosic biomasses (Shi et al. 2008; Kumar and Wyman 2009a). An effective delignification of various feedstocks was reported by fungus *Ceriporiopsis subvermispora* in the combined action of manganese peroxidase and laccase (Wan and Li 2012). A glucose yield of 24.2–56.5 % was reported during enzymatic hydrolysis which was 2–3 folds higher than those of the raw materials. Biological pretreatment of rice husks by fungus *Phanerochaete chrysosporium* resulted in 44.7 % reducing sugars (Potumarthi et al. 2013). Biological treatments of wheat straw by solid state and submerged fermentations in the presence of white-rot basidiomycetes such as *Bjerkandera adusta*, *Fomes fomentarius*, *Ganoderma resinaceum*, *Irpex lacteus*, *Phanerochaete chrysosporium*, *Trametes versicolor*, *Euc*-1 and *Lepista nuda* were evaluated and *T. versicolor* for enzymatic hydrolysis of holocellulose proved better strain compared to others (Pinto et al. 2012). The treatment of hardwood and softwood was also found to be effective with *Streptomyces griseus* (Saritha et al. 2012).

It has been observed that performing saccharification and fermentation processes at high-substrate concentration may increase the concentration of inhibitors (furan derivatives and phenolic compounds). Treatment with enzymes such as laccases has been suggested to prevent production of such inhibitors (Alvira et al. 2010). Martins et al. (2013) showed that recovery of phenolic compounds in the leaves of *Larrea tridentata* was 33 % more in combination of biological treatment followed by methanol extraction compared to methanol extraction alone. Some other advantages of biological pretreatments are: low-capital cost, low energy requirement, no chemicals requirement, and mild environmental conditions (Table 1). However, the main drawback to develop biological methods is that the rate of hydrolysis is very low (Sun and Cheng 2002). There is need to keep on testing more and more isolates such as basidiomycetes fungi for their ability to delignify the plant material quickly and efficiently.

## Conclusions and future perspectives

The various pretreatment technologies for lignocellulosic biomass have been described to improve ethanol production. A major bottleneck in this technology is the presence of lignin which is a major inhibitor of hydrolysis of cellulose and hemicellulose. This has led to extensive research in the development of various pretreatment processes. These processes are based on physical, chemical and biological principles. Chemical and thermo-chemical are currently the most effective and include the most promising technologies for industrial applications. One important point that emerges is that no treatment technology offers 100 % conversion of biomass into fermentable sugars. There is always a loss of biomass, which affects the final yield and increases the cost of finished product, i.e., biofuel. Although pretreatment of lignocellulosic biomass with combination of two or more pretreatment processes has shown promising results, we still feel that there is a need for extensive research in this area so that either a new efficient treatment process is developed or an existing process is upgraded to give promising results. Predictive models will enable the selection, design, optimization, and process control pretreatment technologies that match biomass feedstock with the appropriate method and process configuration.
